# Selective Capturing of the CO_2_ Emissions Utilizing Ecological (3-Mercaptopropyl)trimethoxysilane-Coated Porous Organic Polymers in Composite Materials

**DOI:** 10.3390/polym16131759

**Published:** 2024-06-21

**Authors:** Mohammed G. Kotp, Shiao-Wei Kuo

**Affiliations:** Centre of Functional Polymers and Supramolecular Materials, Department of Materials and Optoelectronic Science, College of Engineering, National Sun Yat-Sen University, Kaohsiung 80424, Taiwan; d09310008@nsysu.edu.tw

**Keywords:** porous organic polymers, nanocomposites, mercapto functionalization, CO_2_ capture, Clausius–Clapeyron equation

## Abstract

Capturing carbon dioxide (CO_2_) is still a major obstacle in the fight against climate change and the reduction of greenhouse gas emissions. To address this problem, we employed a simple Friedel–Crafts alkylation to investigate the effectiveness of porous organic polymers (POPs) based on triphenylamine (TPA) and trihydroxy aryl terms derived from chloranil (CH), designated as TPA-CH POP. We then treated the TPA-CH POP with (3-mercaptopropyl)trimethoxysilane (3-MPTS), forming a TPA-CH POP-SH nanocomposite to enhance CO_2_ capture. Utilizing FTIR, solid-state NMR, SEM, TEM, along with XPS techniques, the molecular makeup, morphological characteristics, as well as physical features of TPA-CH POP and the TPA-CH POP-SH nanocomposite were thoroughly explored. Upon scorching to 800 °C, the TPA-CH POP-SH nanocomposite demonstrated more thermal durability over TPA-CH POP, achieving a char yield of up to 71.5 wt.%. The TPA-CH POP-SH nanocomposite displayed a 2.5-times better CO_2_ capture, as well as a comparable adsorption capacity of 48.07 cm^3^ g^−1^ at 273 K. Additionally, we found that the TPA-CH POP-SH nanocomposite exhibited an improved CO_2_/nitrogen (N_2_) selectivity versus the original TPA-CH POP. Typical enthalpy changes for CO_2_ capture were somewhat increased by the 3-MPTS coating, indicating greater binding energies between CO_2_ molecules and the adsorbent surface. Our outcomes demonstrate that a TPA-CH POP composite coated with MPTS is a viable candidate for effective CO_2_ capture uses. Our findings encourage the investigation of different functional groups and optimization strategies.

## 1. Introduction

The levels of carbon dioxide (CO_2_) are a significant factor in climate change and the phenomenon of global warming. Reducing the negative impacts of CO_2_ emissions requires the use of efficient carbon capture and storage (CCS) and utilization (CCU) technologies [1,2,3,4]. Adsorption-based processes have drawn a lot of interest among the several CCS and CCU technologies because of its promise for high efficiency, relatively little energy use, and minimal ecological impact [5,6,7,8,9,10,11]. The research has recently centred on the creation of novel materials for CO_2_ capture [12,13]. Significantly, because of their considerable surface area that enables the adsorption of massive amounts of CO_2_ molecules, porous organic polymers (POPs) have emerged as potential materials for capturing carbon dioxide (CO_2_) [14,15,16,17,18,19,20,21,22,23,24,25,26,27,28,29,30]. 

Additionally, they provided customizable porosity, meaning that CO_2_ can be selectively captured over other gas molecules by adjusting the size and shape of the holes. Moreover, the POP structure can be modified by adding functional groups to improve its selectivity and capability for CO_2_ collection [19]. Triphenylamine (TPA)-based POPs have a special appeal among POPs because of their potential to tolerate high levels of heat, which are frequently encountered in industrialized CO_2_ capture operations. They also do not break down easily when other gasses are present [31,32,33,34,35,36,37]. Notably, TPA’s steric and electronic properties are flexible enough to affect CO_2_ capture effectiveness [38,39,40]. Since the POP architecture has many tiny pores that allow for CO_2_ adsorption, these POP-based TPAs are projected to show significant CO_2_ collection power [41,42,43,44]. Alternatively, nitrogen atoms from the TPA subunits can engage strongly with CO_2_ molecules via favourable electrostatic forces [45].

In particular, well-designed POPs including TPA and chloranil (CH) subunits, such as TPA-CH POP, can still have CO_2_ capture power which can be increased by post-synthetic adjustments. The use of 3-mercaptopropyltrimethoxysilane (3-MPTS) is expected to be relevant in this situation. Extra mesopores, or slightly bigger pores, can be added to the TPA-CH POP architecture by incorporating 3-MPTS to improve the surface area accessible for CO_2_ adsorption. Additionally, the thiol groups (-SH) in 3-MPTS have the ability to form hydrogen bonds with CO_2_ molecules, enhancing the contact and increasing the capacity for CO_2_ collection. Thus, the creation of TPA-CH POP in nanocomposites that have been altered with 3-MPTS holds immense promise for the generation of CO_2_ capture substances that are more effective. Seeking improvements in environmentally friendly technologies is a means through which we can reduce global warming.

The strategic use of a 3-MPTS coating represents a notable modification that has been found to boost the adsorption capabilities of numerous materials [46]. 3-MPTS is a flexible silane coupling agent which can be utilized to alter a material’s surface characteristics, including adding new functional groups, enhancing adherence, and making a substance more hydrophobic [47,48]. Further, the combination of 3-MPTS with silica improves the adsorption qualities of a range of specific chemicals, including selectivity and capacity [48,49,50]. These scenarios show how the efficiency of the material itself can be enhanced by including 3-MPTS, resulting in increased selectivity, capacity, as well as durability throughout adsorption processes. Thus, we anticipate that by merging the inherent qualities of TPA-CH POP along with the specific improvements provided by 3-MPTS, chemists will be able to develop cutting-edge materials with exceptional CO_2_ capture capabilities, opening the door to a brighter tomorrow. 

**Scheme 1:**
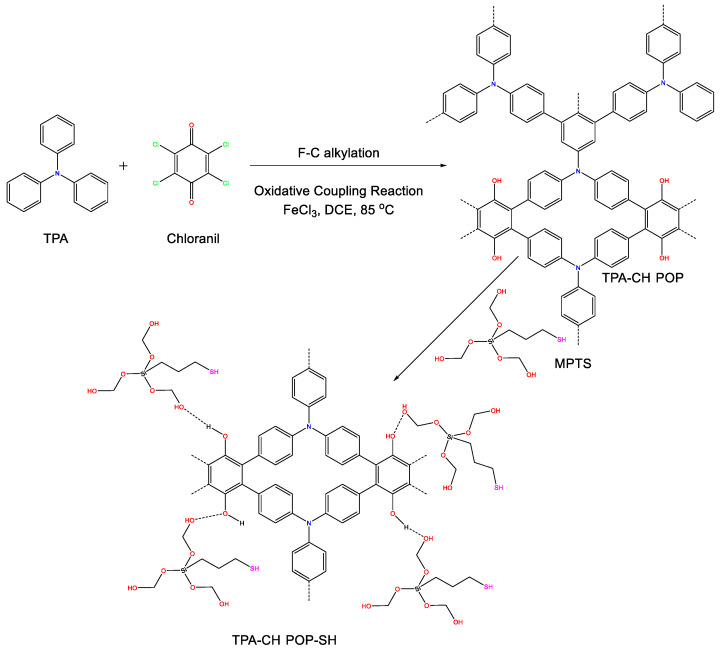
Synthesis of TPA-CH POP and the TPA-CH POP-SH nanocomposite.

As shown in Scheme 1, we created the TPA-CH POP used in this research by utilizing Friedel–Crafts alkylation involving TPA and CH. Additionally, we used the coprecipitation approach to create a nanocomposite that included the TPA-CH POP as well as 3-MPTS. FTIR, SSNMR, and XPS analyses were used to study the pristine TPA-CH POP and the TPA-CH POP-SH nanocomposite. A surface area of up to 850 m^2^ g^−1^ and a thermal stability of 71.5 wt.% were achieved using these novel materials. Additionally, we looked into the CO_2_ absorption capabilities of the pure TPA-CH POP as well as the TPA-CH POP-SH nanocomposite. We investigated how the 3-MPTS coating affects the adsorption characteristics of the TPA-CH POP-SH composite, which was 2.5 times higher compared to untreated TPA-CH POP. To the best of our knowledge, this pioneering study is the first to explore the application of MPTS-coated polymers for CO_2_ capture. We also calculated the standard enthalpy adjustments and CO_2_/N_2_ selectivity. Overall, this research emphasizes how important new materials as well as modifications are for efficient CO_2_ capture. The TPA-CH POP composites and other porous organic materials can perform better in adsorption activities after 3-MPTS treatment. The results of this research add to the continuous search for practical and long-lasting ways to reduce CO_2_ emissions. 

## 2. Materials and Methods

### 2.1. Materials

Reagents including dichloroethane (DCE, 99%) and anhydrous iron chloride (anhydrous FeCl_3_) were provided by Sigma Aldrich (Saint Louis, MO, USA). The triphenylamine was provided by Alfa Aesar (Ward Hill, MA, USA. The chloranil (CH powder) was given by TCI (Portland, OR, USA). IOTA CORPORATION LTD. (Bengbu, China) provided the 3-mercapto-propyltriethoxysilane (3-MPTS). Thermo Fisher, (Taipei City, Taiwan), supplied the hydrochloric acid (35%) as well as ultrapure butanol, ethanol, and methanol, as well as toluene. It should be emphasized that the water was double-distilled, and all of the solvents used in our study were analytical grade and therefore did not need to be further treated.

### 2.2. Design of TPA-CH POP

TPA (0.98 g, 4.1 mmol) and anhydrous FeCl_3_ (1.97 g, 0.012 mmol) were added to 50 mL of pure 1,2-dichloroethane along with 4.1 mmol of CH powder (1 g) for the reaction. After being electromagnetically stirred for 10 h at room temperature, the mixture was heated to 100 °C and held at that temperature for four days straight. The elastic material was separated during this process. The resultant material was purified, washed with methanol, and then reconstituted using methanol. In the end, a yellow-green substance was extracted and allowed to dry under a vacuum at 100 °C for an entire day (yield: 93%). 

### 2.3. Design of the TPA-CH POP-SH Nanocomposite

The constructed TPA-CH POP was effectively decorated with thiol clusters using the methods described by Liu et al. [51], albeit with small modifications: 1.5 g of TPA-CH POP was mixed with 25 mL of dry toluene. Following that, the mixture was refluxed and electromagnetically stirred over a N_2_ atmosphere for an hour. After that, 1.5 mL of 3-MPTS was added to the mixture and refluxed continuously for two additional days. The result was allowed to settle for a period of three hours, and then it was removed, washed with ethanol, and dried at 80 °C for around five hours. 

### 2.4. Gas Uptake Studies

#### 2.4.1. Isotherms

The CO_2_ as well as N_2_ utilization isotherms of TPA-CH POP and the TPA-CH POP-SH nanocomposite were measured at 298 K as well as 273 K, utilizing an ASAP 2020 gaseous intake analyser. Typically, the prescribed materials (ca. 30–90 mg) were dried for 5 h over degassing processes at 100 °C. Following this, the materials were packed into analyser units to figure out the gas absorption isotherms at two different temperatures of 273 K and 298 K, using an ongoing administration of exceptionally purified gases of CO_2_ or N_2_ (up to close to 1 bar). For our comparison research, we utilized the CO_2_ and N_2_ capture estimates at observed pressures of *P*/*P_o_* = 1.

#### 2.4.2. The Selective Assessments

The selectivity assessments of TPA-CH POP and the TPA-CH POP-SH nanocomposite across CO_2_/N_2_ were determined utilizing Henry’s formula, as described earlier. Significantly, the constant of Henry’s law takes into account the slope of the gas utilization loop at a diminished level of 0.1 bar. 

## 3. Results

### 3.1. Structure and Characteristics of TPA-CH POP

**Figure 1:**
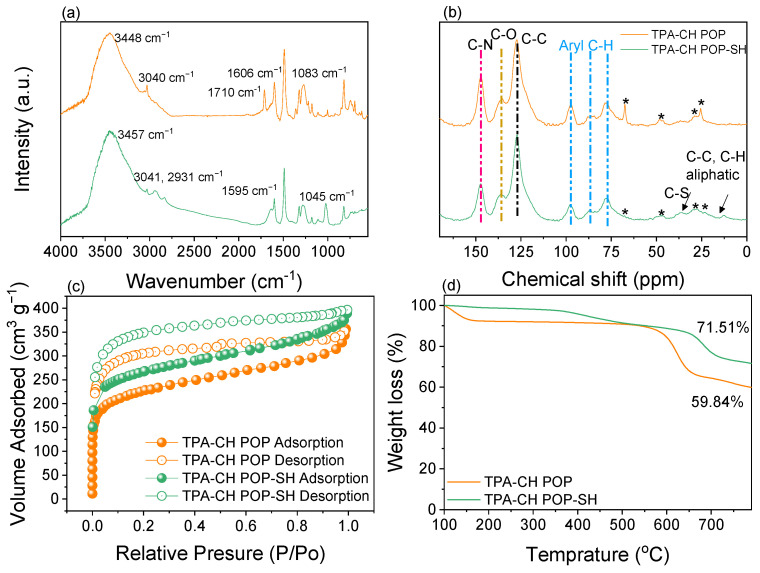
FTIR (**a**), SSNMR (**b**), BET (**c**), and TGA (**d**) profiles of the TPA-CH POP and the TPA-CH POP-SH nanocomposite. The * signs of (d) have been revealed to the machine.

Utilizing a combat ion of CH with an aromatic molecule of TPA via a Friedel–Crafts alkylation technique, the current CH-based POP (TPA-CH POP) was created in a measurable yield (Scheme 1). Notably, a considerable level of crosslinking was shown by the TPA-CH POP’s inability to dissolve in common solvents such as acetone, ethanol, methanol, and tetrahydrofuran [52]. CH has quinone groups which can be easily converted into hydroxyl groups in this reaction media; consequently, our TPA-CH POP can offer hydroxyl sites instead [53]. Notably, the presence of C=O signals from the CH units is clearly evident in the FTIR spectrum of our TPA-CH POP at 1710 cm^−1^, indicating their presence to a certain degree. Using solid-state ^13^C cross-polarization (CP)/magic angle spinning (MAS) NMR spectroscopies and Fourier transform infrared (FTIR) spectroscopy, the chemical architecture of the developed TPA-CH POP was examined. First, the TPA-CH POP’s FTIR spectrum (Figure 1a) demonstrates the complete disintegration of the strong signal at 600 cm^−1^, which is caused by vibrating C-Cl components and confirms the linkage throughout the building blocks [54]. Moreover, the TPA-CH POP exhibited FTIR signals at 3448, 3040, 1606, and 1083 cm^−1^, which correspond to stretching vibrations of C-OH, C-H (aromatic), C=C, and C-O, respectively [19,55]. Notably, the presence of C=O signals from the CH units is evident in the FTIR spectrum of the TPA-CH POP at 1710 cm^−1^ (Figure 1a), indicating its presence to a certain degree. Furthermore, the existence of nitrogen in POPs might cause structural defects at carbon edge locations of unsaturated carbon atoms that are highly reactive to physically adsorbed oxygen, which can be found in various states [19]. These signals show that TPA-CH POP was effectively synthesized. Figure 1b displays the solid-state ^13^C NMR (SSNMR) results for the TPA-CH POP, which revealed distinct traces in the 109–102 ppm area, indicating the aromatic C-H portions, and a distinct pattern at 143.5 ppm, which is linked to the carbon nuclei of C-O across the TPA-CH POP. Additionally, the TPA-CH POP showed an additional signal attributed to the C-N parts at 118.4 ppm [56]. 

**Figure 2:**
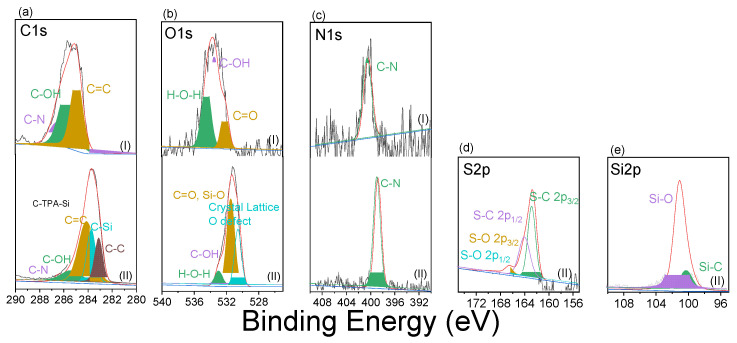
High-resolution XPS scans of C1s (**a**), O1s (**b**), N1s (**c**), S2p (**d**), and Si2p (**e**) of the TPA-CH POP (I) and the TPA-CH POP-SH nanocomposite (II). (The grey, and pink colours reveal to the original and fitted XPS data).

To better illustrate the fundamental structure of TPA-CH POP surfaces and their respective electronic states, we applied the XPS approach. Within this setting, the TPA-CH POP solid’s chemical pattern was confirmed by clearly visible high-resolution XPS signals of C1s, O1s, and N1s (Figure 2 and Figure S1). A total of four bands (Figure 2a) arose from the deconvolution of the C1s impulses from the TPA-CH POP sample. These bands correspond to different types of carbon and include sp^2^ carbon particles (C=C, 284.8 eV), sp^2^ carbons linked to hydroxy oxygen (C-OH, 285.9 eV), sp^2^ carbons (C-N, 286.3 eV), and sp^2^ keto-carbon C=O units (287.1 eV). The TPA-CH POP specimen’s XPS spectrum showed three oxides at 534.4, 533.5, and 532.2 eV (Figure 2b), which were attributed to hydrated oxygen, C-OH, and -C=O species, respectively. Furthermore, the N1s response, which, for C-N molecules, corresponds to a single-molecule peak at 400.1 eV, is shown in the TPA-CH POP spectrum (Figure 2c). The nitrogen adsorption/desorption isotherm at 77 K was used to calculate the total surface area connected to the manufactured TPA-CH POP (Figure 1c). This isotherm showed a sharp rise at relatively low pressure, indicating that it is microporous (Figure 1c) [57]. It also showed that TPA-CH POP has extremely specific surfaces up to 822 m^2^ g^−1^ (Figure 1c), with this surface area likely surpassing that of known porous polymers [22,58]. 

**Figure 3:**
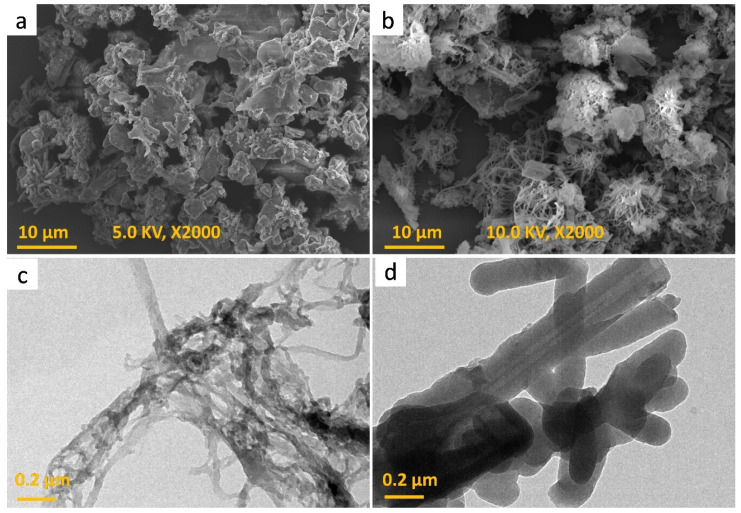
SEM (**a**,**b**), and TEM (**c**,**d**) images of the TPA-CH POP and the TPA-CH POP-SH nanocomposite.

Furthermore, abrupt desorption processes arose from a *P*/*P_o_* of 0.5 to 0.45, revealing N_2_ rupture and demonstrating that the mesopores have an ink-bottle arrangement. In reality, the mesopores of the TPA-CH POP correspond to elasticity-related deformation as well swells, resulting in gas absorption [19,57]. POPs are well-known for having high thermal strengths; the thermal gravimetric analysis (TGA) indicated that the TPA-CH POP exhibited an extremely high degree of thermal endurance under inert conditions. The degradation temperature (*T*_d10_) of the TPA-CH POP was 540 °C, as indicated in Figure 1d, leading to an outstanding char output of 59.8 wt.%. The remarkable thermal stability of the TPA-CH POP suggests a robust π-π stacking relationship between its layers [59]. The physical traits, chemical makeup, and sizable surface areas of TPA-CH POP render it a viable 3-MPTS wrapping substrate. Field emission scanning electron microscopy (FE-SEM) and high-resolution transmission electron microscopy (HR-TEM) were employed to investigate the morphological features of the TPA-CH POP. According to Figure 3a, the FE-SEM scan showed that this TPA-CH POP became somewhat agglomerated. However, a coaxial structure could be observed in the TPA-CH POP’s TEM image (Figure 3c). The coaxial structure of the TPA-CH POP was attributed to the planarity CH units [60,61].

### 3.2. Structure and Characteristics of TPA-CH POP-SH Nanocomposite

We developed a TPA-CH POP-SH nanocomposite using ecologically friendly decoration (no hazardous reduction chemicals were used). Uniquely, the hydroxyls produced by hydrated 3-MPTS were anticipated to form hydrogen links with those of TPA-CH POP molecules throughout the refluxing operation (Scheme 1). Particular functional groups were present in the TPA-CH POP-SH nanocomposite, according to the FTIR assessments. Following the modification with 3-MPTS, a chemical reaction appeared to have occurred based on the changes in peak locations between the TPA-CH POP-SH nanocomposite versus the TPA-CH POP (Figure 1a). The TPA-CH POP-SH nanocomposite exhibited a peak shift of 3457 cm^−1^ compared to 3448 cm^−1^ in the pristine TPA-CH POP. This shift is indicative of the existence of hydroxyl groups, as shown by the stretching vibration of O-H bonds. However, the aromatic C-H stretching vibration was linked to the peak at 3041 cm^−1^ in both samples, indicating the existence of aromatic chains in our matrixes [62]. Otherwise, the TPA-CH POP-SH nanocomposite’s peak at 2931 cm^−1^, which may be related to the 3-MPTS modifier’s presence, showed the stretching vibration of aliphatic C-H bonds. Additionally, the TPA-CH POP-SH nanocomposite’s peak at 1595 cm^−1^, which is slightly different from the pristine TPA-CH POP’s peak at 1606 cm^−1^, was associated with the aromatic rings’ C=C stretching vibration, indicating the existence of aromatic structures and the connection between these two components. Moreover, the TPA-CH POP-SH nanocomposite’s peak at 1045 cm^−1^, which relates to Si-O-Si asymmetric stretching vibrations, suggests the existence of siloxane units from 3-MPTS [63]. Both the TPA-CH POP-SH nanocomposite along with the original TPA-CH POP exhibited peaks in their SSNMR spectrum (Figure 1b) at 118.4 ppm and 143.5 ppm, and a broad range from 109 and 102 ppm. However, the TPA-CH POP-SH nanocomposite also displayed further peaks at 37.5 ppm and 12.5 ppm. In line with earlier research, the peak at 143.5 ppm was most likely caused by the triphenylamine (TPA) moiety. The carbon atoms in the TPA’s aromatic rings and the CH units in the pure TPA-CH POP may be responsible for the large range of peaks between 109 and 102 ppm. The carbon atoms in the C-N could be the cause of the peak at 118.4 ppm. The inclusion of the 3-MPTS modifier may be the cause of two more peaks in the composite at 37.5 ppm and 12.5 ppm (Figure 1b). The carbon atoms in the methylene units (-CH_2_-) of MPTS may be the cause of the peak at 12.5 ppm, while the carbon atoms in the thiol groups (C-S) of 3-MPTS may be the cause of the peak at 37.5 ppm [64]. These peaks are consistent with the FTIR results and indicate that 3-MPTS successfully changed the porous polymer. 

The chemical composition and electronics of the compounds can be predicted from the X-ray Photoelectron Spectroscopy (XPS) patterns of the TPA-CH POP as well as the composite after treatment with 3-MPTS. As previously stated, the C1s signals for the TPA-CH POP separated into four parts, including the sp^2^ carbon particles (C=C, 284.8 eV), sp^2^ carbons connected to hydroxy oxygens (C-OH, 285.9 eV), sp^2^ carbons (C-N, 286.3 eV), and sp^2^ keto-carbons (C=O units, 287.1 eV). Following the 3-MPTS coating, the C1s signals split into five components at binding energies of 286.3, 285.5, 284.9, 283.7, and 283.1 eV, corresponding to C-N, C-OH, C=C, C-Si, and C-C. The corresponding areas under the peaks were 90.56%, 12.89%, 49.02%, 23.49%, and 20.08%, respectively (Table S1). The emergence of additional carbon-related peaks belonging to C-Si and C-C bonds, which are typical for MPTS, indicates the effective integration of MPTS into the TPA-CH POP matrix. Furthermore, the XPS data confirm that there are C-N, C-OH, and C=C bonds in both the TPA-CH POP and the composite, showing that the original TPA-CH POP composition was retained even after MPTS treatment. The TPA-CH POP-SH nanocomposite’s O1s XPS signal was deconvolved into O-H, O-C, (C=O or O-Si), and a crystal lattice O defect at 534.4, 533.4, 532.1, and 530.7 eV, respectively (Figure 2b). This process yielded significant data regarding the chemical environment of the oxygen atoms in the composite. The existence of hydroxyl groups was indicated by the O-H signal at 534.4 eV, and the presence of oxygen in carbon bonds was suggested by the O-C signal at 533.4 eV. Carbonyl groups (C=O) or silicon bond oxygen (O-Si) could be the source of the C=O or O-Si signal at 532.1 eV. The presence of oxygen vacancies or impurities in the composite was indicated by the crystal lattice O defect at 530.7 eV. The O1s signal’s deconvolution revealed several oxygen-containing functional groups and imperfections, offering insights into the composite’s surface chemistry. The O-Si linkages imply that silicon from the MPTS modifier was incorporated onto the TPA-CH POP matrix’s surface. Furthermore, the reactivity and characteristics of the composite material may be impacted by the existence of oxygen vacancies or flaws. The N1s at 400.4 eV seen in the TPA-CH POP-SH nanocomposite (Figure 2c) may be the result of nitrogen atoms that were retained in the TPA-CH POP matrix even after the 3-MPTS coating. It appears that the MPTS coating had little impact on the nitrogen atoms in the TPA-CH POP matrix because the N1s spectra did not show multiple peaks. The S2p signal obtained from the XPS analysis of the TPA-CH POP-SH nanocomposite deconvoluted into coupled components such as S-O 2p3/2 and S-O 2p1/2 at 168.5 and 166.5 eV, respectively, and S-C 2p3/2 and S-C 2p1/2 at 163.8 and 162.6 eV, respectively (Figure 2d). The S-O signals suggest the presence of sulphur in oxygen-containing functional groups, while the S-C signals indicate the presence of sulphur in carbon-containing functional groups. The presence of S-O and S-C bonds in the TPA-CH POP-SH nanocomposite could be attributed to the incorporation of sulphur elements from the 3-MPTS modifier onto the surface of the TPA-CH POP. From the deconvolution of XPS signal for the Si atom, we detected coupled states of Si2p of the Si-O and Si-C at 101.15 and 100.05 eV, respectively (Figure 2e), with a high inclusion percentage of the former, as shown in Table S1. 

The surface area of the composite material can be indirectly affected by the surface modification of nanoparticles with molecules like 3-MPTS, which has been demonstrated to improve compatibility with polymer matrices and modify their surface attributes [65]. Additionally, surface-initiated controlled radical polymerization, which involves the use of polymer brushes for surface modification, has been reported as a method to tailor the chemical and physical properties of interfaces, potentially affecting the surface area of the modified polymers [66]. The TPA-CH POP’s surface area was calculated to be 822 m^2^ g^−1^. The surface area of the TPA-CH POP rose to 850 m^2^ g^−1^ after treatment with 3-MPTS (Figure 1c). The 3-MPTS coating’s modification of the porous polymer’s surface chemistry was probably what caused this increase in surface area. This might boost the quantity of active sites that are available for adsorption and improve the pores’ usability. It is crucial to remember that the surface area was only slightly increased, and that the surface area may also be impacted by additional variables including the pore size distribution and the shape of the porous polymer [67].

The MPTS modifier was responsible for the char yield increase from 59.8% for the pristine TPA-CH POP to 71.5% upon coating with 3-MPTS (Figure 1d). It is well known that 3-MPTS contains silicon. As the composite degrades thermally, the silicon-containing compounds create a layer of silicon carbide that acts as protection and adds to the char residue [68]. This behaviour is frequently observed in materials that have been treated with organosilanes; compounds that include silicon promote the development of char during thermal decomposition, which raises the char yield in TGA analyses [69]. Because silicon-containing compounds are known to enhance char formation throughout thermal degradation, the rise in char yield observed in the TGA study of the TPA-CH POP-SH nanocomposite following coating with 3-MPTS makes sense. A higher char yield with the 3-MPTS coating indicates better resistance to oxidative degradation as well as improved thermal durability [70]. This discovery is consistent with the idea that the increased char production is caused by silicon-containing compounds forming a protective layer of silicon carbide during thermal breakdown. When the TGA and BET investigation results are combined, it provides a comprehensive understanding of the material’s behaviour at high temperatures and clarifies the underlying mechanisms driving the changes brought about by 3-MPTS. 

The surface chemistry and thermal stability of the composite material can be better understood by combining the analysis of the XPS results with those of the BET and TGA data. One can determine that the functional groups have been utilized without changing the surface area, for example, if the BET results show no appreciable changes in the surface area but the XPS results suggest an increase in the intensity of some functional groups following treatment with MPTS. Conversely, if the TGA findings indicate that the material’s thermal stability has improved following MPTS treatment, it can be concluded that the 3-MPTS has improved the material’s thermal stability. 

**Figure 4:**
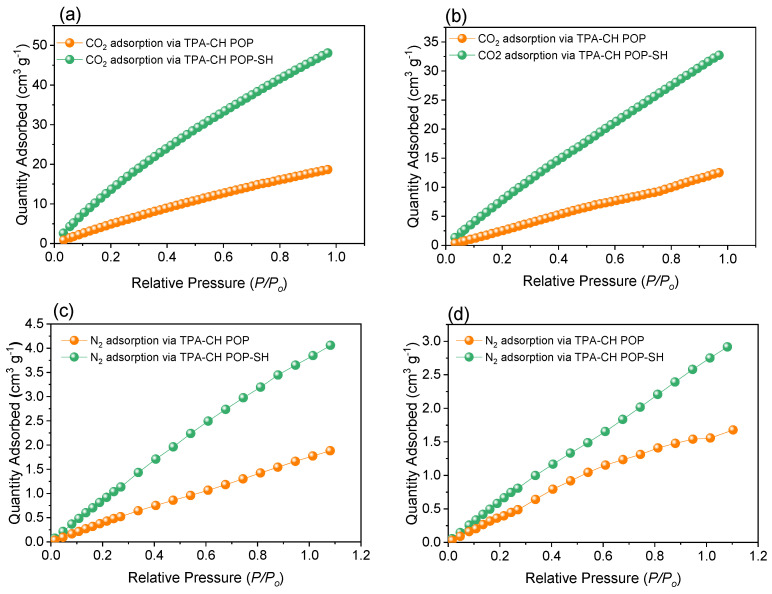
The uptake profiles of CO_2_ (**a**,**b**) and N_2_ (**c**,**d**) by the TPA-CH POP and the TPA-CH POP-SH nanocomposite, respectively, at two different temperatures of 273 K (**a**,**c**) and 298 K (**b**,**d**).

The pure TPA-CH POP and the TPA-CH POP-SH nanocomposite exhibited aggregated particles with minimal coaxial topologies in their scanning electron microscopy (SEM) visualizations (Figure 4a,b). We may assume that the addition of 3-MPTS altered the TPA-CH POP’s surface, which induced the particles to interact with one another and form aggregates. Alternatively, this relates to the fact that the existence of compounds like 3-MPTS that include silicon encourages inter-particle bonding through van der Waals forces, which results in the aggregation of particles. On the other hand, as the solution’s viscosity increases during the solvent evaporation process, capillary forces and decreased mobility lead to a tendency for the particles to agglomerate. Coaxial morphology was observed in the TEM images of the TPA-CH POP nanocomposite after coating with 3-MPTS, as well as in the images of the virgin TPA-CH POP (Figure 3c,d). This observation indicates that when the polymer is combined with 3-MPTS, the self-assembled nature of the TPA-CH POP enables the production of coaxial structures. These coaxial structures’ core–shell architecture develops spontaneously as a result of 3-MPTS’s selective adsorption onto the TPA-CH POP particles’ surfaces. 

### 3.3. Gas Uptake Investigations

Technologies for capturing carbon dioxide (CO_2_) are crucial for minimizing greenhouse gas emissions and the effects of human-caused climate change [71,72,73]. Here, we examined how a new organosilane TPA-CH POP-SH nanocomposite can be used to improve CO_2_ collection capacities. The pure TPA-CH POP and the MPTS-coated TPA-CH POP composite had their CO_2_ uptake capabilities assessed at 273 K and 298 K throughout this investigation. For the pristine TPA-CH POP and the TPA-CH POP-SH nanocomposite, the CO_2_ uptake values at 273 K were determined to be 18.66 cm^3^ g^−1^ and 48.07 cm^3^g^−1^, respectively (Figure 4a and Table S2). 

Likewise, at 298 K, the CO_2_ uptake values for the pristine TPA-CH POP and the TPA-CH POP-SH nanocomposite (Figure 4b) were found to be 12.5 cm^3^ g^−1^ and 32.69 cm^3^ g^−1^, respectively. The TPA-CH POP-SH nanocomposite showed a comparable performance to that of previously reported materials, as shown in Table S3. There are multiple reasons for the significant increase in CO_2_ capture abilities that were noted for the MPTS-coated TPA-CH POP composite. First, the TPA-CH POP composite’s surface area may increase as a result of the MPTS coating, giving CO_2_ molecules a greater chance to interact and improving CO_2_ adsorption [74]. Second, the MPTS coating’s introduction of extra functional groups comprising silicon, oxygen, and carbon produces more CO_2_ adsorption sites, improving the composite’s capacity to absorb CO_2_ [75]. Thirdly, the MPTS coating’s enhancement of the microporous architecture guarantees quick mass transfer as well as the successful use of available sites for adsorption [76]. Ultimately, the greater interactions between the composite and CO_2_ resulted in an improved CO_2_ capture capacity due to the improved thermodynamic affinity between the composite and CO_2_ molecules brought about by the addition of additional functional groups and the development of a microporous structure [77]. Our results show that, in comparison to the pure TPA-CH POP, the MPTS-coated TPA-CH POP composite has significantly improved CO_2_ collection capabilities. The development of extra functional groups, the enhancement of the microporous network, the higher thermodynamic affinity, and the increase in surface area were all responsible for the notable improvements in CO_2_ collection efficiency [78]. Our research demonstrates the potential use of organosilane-coated TPA-CH POP composites in CO_2_ capture technologies to minimize the effects of anthropogenic climate change and limit greenhouse gas emissions.

**Figure 5:**
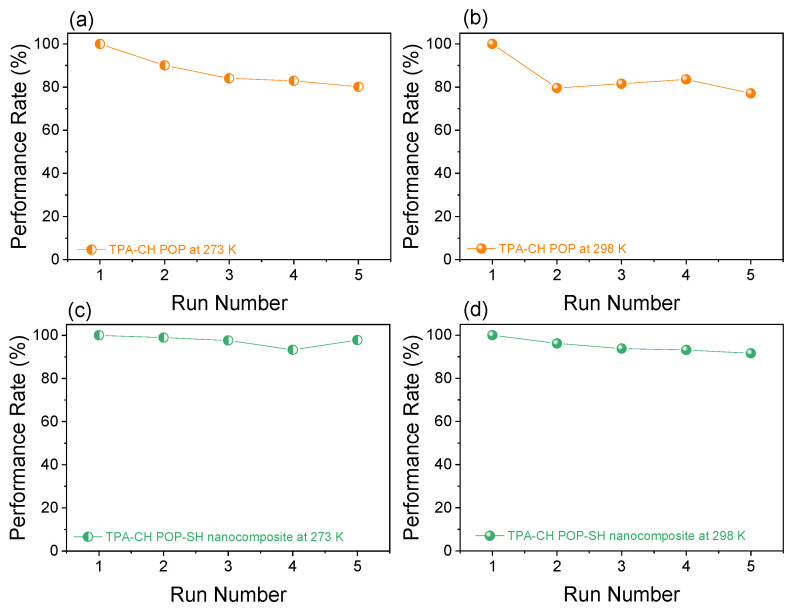
Recyclability of the TPA-CH POP (**a**,**b**) and the TPA-CH POP-SH nanocomposite (**c**,**d**) for CO_2_ uptake at 273 K and 298 K, respectively, for 5 consecutive cycles.

The CO_2_ uptake efficiency was tested during five consecutive cycles at 273 K and 298 K in order to assess the TPA-CH POP and the modified TPA-CH POP-SH nanocomposite’s long-term applicability (Figure 5). Over the course of all cycles, the CO_2_ uptake values stayed largely consistent, suggesting that the pristine TPA-CH POP and the TPA-CH POP-SH nanocomposite both had good reversibility and high recyclability. The CO_2_ uptake values for the original TPA-CH POP and the TPA-CH POP-SH nanocomposite at 273 K were 18.66 cm^3^ g^−1^ and 48.07 cm^3^ g^−1^, respectively, as illustrated in Figure 5. At 298 K, the equivalent values were 12.50 cm^3^ g^−1^ and 32.69 cm^3^ g^−1^, respectively. The CO_2_ uptake values for each substance stayed mostly constant throughout the course of the five cycles, indicating their remarkable reversibility and great recyclability. According to these results, there is a lot of potential for the TPA-CH POP-SH nanocomposite in real-world CO_2_ capture applications that try to lower greenhouse gas emissions and diminish the effects of human-caused climate change. Furthermore, the TPA-CH POP-SH nanocomposite is a sustainable and affordable choice for large-scale CO_2_ capture systems due to its excellent recyclability. 

The TPA-CH POP-SH nanocomposite had good recyclability, as evidenced by the consistency of its CO_2_ uptake values over the course of five cycles. This suggests that the material’s CO_2_ capture capabilities are not significantly compromised during use. Importantly, the TPA-CH POP-SH nanocomposite’s superior recyclability provides compelling evidence for its application in CO_2_ capture systems designed to reduce greenhouse gas emissions and the effects of human-driven climate change.

**Figure 6:**
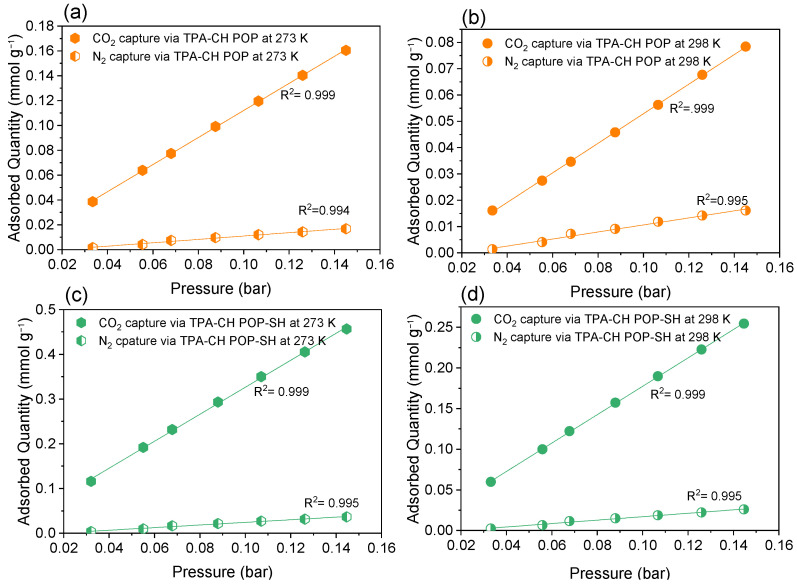
Gas uptake selectivity at 273 K and 298 K of the TPA-CH POP (**a**,**b**) and the TPA-CH POP-SH nanocomposite (**c**,**d**), respectively, employing the ratios of the Henry’s law constant calculated from the initial slopes of the single-component gas adsorption isotherms collected at low pressures up to 0.16 bar.

By utilizing the slopes of the gas uptake isotherms at lower pressures of up to 0.15 bar at temperatures of 298 K and 273 K, the CO_2_/N_2_ selectivity results were obtained using Henry’s model constant (Figure 6) [79]. It was discovered that the TPA-CH POP-SH nanocomposite had a better selectivity for CO_2_ over N_2_ than the pristine TPA-CH POP. The TPA-CH POP-SH nanocomposite’s CO_2_/N_2_ selectivity at 273 K was 11.869, compared to 9.925 for the virgin TPA-CH POP. The TPA-CH POP-SH nanocomposite exhibited a CO_2_/N_2_ selectivity of 11.27 at 298 K, whereas the virgin TPA-CH POP demonstrated a selectivity of 7.453.

These findings imply that the 3-MPTS coating improves the TPA-CH POP composite’s selectivity towards CO_2_ versus N_2_. Due to the MPTS coating’s introduction of additional functional groups like Si-O, S-O, and S-C bonds, the TPA-CH POP composite had a greater CO_2_/N_2_ selectivity. These new functional groups give CO_2_ molecules more places to adsorb, increasing the CO_2_ absorption capabilities and improving CO_2_ selectivity over N_2_. This enhanced selectivity is a key factor in the potential application of the TPA-CH POP-SH nanocomposite for efficient CO_2_ capture and separation processes. These results are in accordance with the literature, which emphasizes the significance of selectivity, functional groups, and surface characteristics in influencing the way that composite materials adsorb gases for separation and capture. The TPA-CH POP-SH nanocomposite’s improved CO_2_/N_2_ selectivity lends credence to its potential as a successful adsorbent for CO_2_ capture applications, especially when N_2_ is present. These findings imply that the MPTS coating enhances the TPA-CH POP composite’s adsorption capabilities, indicating that it is a potential material for CO_2_ capture and separation processes.

### 3.4. Isotherms of CO_2_ Uptake and Standard Enthalpy Changes

**Figure 7:**
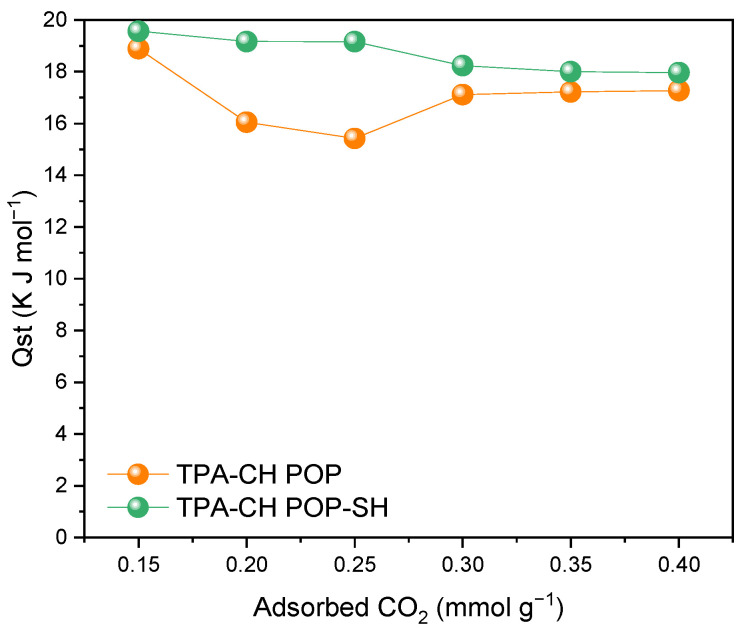
Isosteric heat of CO_2_ uptake at 273 K and 298 K by the TPA-CH POP and the TPA-CH POP-SH nanocomposite.

The pristine TPA-CH POP and the TPA-CH POP-SH nanocomposite had standard enthalpy change (ΔH°) values for CO_2_ adsorption of 18.89 and 17.27 kJ/mol and 19.57 and 17.97 kJ/mol, respectively, at 0.15 bar and 0.17 bar for CO_2_ uptake, respectively, based on the Clausius–Clapeyron equation assumptions (Figure 7) [80]. According to these findings, when compared to the pristine TPA-CH POP, the 3-MPTS coating caused somewhat larger standard enthalpy changes for CO_2_ adsorption. According to this research, the MPTS coating increased the binding energy between CO_2_ molecules and the adsorbent surface, which boosts the TPA-CH POP composite’s total adsorption capacity and selectivity towards CO_2_ over N_2_. It is important to remember that the Clausius–Clapeyron equation is based on the assumption that the system behaves ideally [81]. Notably, when non-ideal behaviours such multilayering, site blocking, and interactions between adsorbed molecules are taken into account, the findings may differ from reality. 

## 4. Conclusions

We used the straightforward Friedel–Crafts and coprecipitation methods to develop TPA-CH POP and a TPA-CH POP-SH nanocomposite, respectively. We used FTIR, SSNMR, and XPS analyses to highlight their chemical characteristics. In addition, the physical characteristics of the material were highlighted based on N_2_ adsorption/desorption isotherms, and TGA, SEM, and TEM analyses. Compared to the pure TPA-CH POP, the TPA-CH POP-SH nanocomposite exhibited a larger surface area and thermal stabilities of up to 850 m^2^ g^−1^ and 71.5 wt.%, respectively. Additionally, the TPA-CH POP-SH nanocomposite results showed how the MPTS coating significantly affects the CO_2_ adsorption ability. Furthermore, a slightly higher standard change in enthalpy for CO_2_ adsorption was observed with the TPA-CH POP-SH nanocomposite, suggesting stronger binding energies between CO_2_ molecules and the adsorbent surface. These findings demonstrate the TPA-CH POP-SH nanocomposite’s promise as a material for effective carbon dioxide capture applications, especially when nitrogen is present. The TPA-CH POP-SH nanocomposite is a strong option for CO_2_ capture applications due to its improved selectivity and adsorption capabilities. Overall, this work highlights how innovative composite materials, like the MPTS-coated TPA-CH POP, could significantly advance carbon capture technology and address greenhouse gas emission-related ecological issues. The knowledge gathered from this study promotes the ongoing quest for novel approaches for reducing climate change and advancing towards a more sustainable future. 

## Data Availability

The original contributions presented in the study are included in the article/Supplementary Material, further inquiries can be directed to the corresponding author.

## Supplementary Materials

The following supporting information can be downloaded at: https://www.mdpi.com/article/10.3390/polym16131759/s1, Characterization instrument details; Figure S1: Wide XPS scan of the TPA-CH POP and TPA-CH POP-SH nanocomposite; Table S1: Fitted data of TPA-CH POP, and TPA-CH POP-SH nanocomposite; Table S2: CO_2_ and N_2_ uptakes, CO_2_/N_2_ selectivity, and isosteric heat of the designed TPA-CH POP and TPA-CH POP-SH nanocomposite; Table S3: Comparison of the TPA-CH POP-SH nanocomposite with previously reported polymers in terms of CO_2_ capture. References [82,83,84,85,86,87,88,89] are cited in the supplementary materials.
