# Supplementary material for: Selective Capturing of the CO2 Emissions Utilizing Ecological (3-Mercaptopropyl)trimethoxysilane-Coated Porous Organic Polymers in Composite Materials
Source: Polymers (Basel). 2024 Jun 21;16(13):1759. doi: 10.3390/polym16131759 (PMC11243962; doi:10.3390/polym16131759)
Supplement: Supplementary file 1 [file polymers-16-01759-s001.zip › polymers-3049500-supplementary.pdf]

Supporting Information

Selective Capturing of the CO_2 Emissions Utilizing Ecological (3-Mercaptopropyl)trimethoxysilane Coated Porous Organic Polymers in Composite Materials

Mohammed G. Kotp and Shiao-Wei Kuo*

Department of Materials and Optoelectronic Science, Center for Functional Polymers and Supramolecular Materials, National Sun Yat-Sen University, Kaohsiung, 80424, Taiwan

Corresponding Author:

E-mail: kuosw@faculty.nsysu.edu.tw (S. W. Kuo)

Characterizations

Fourier transform infrared (FTIR)

Contemporary KBr discs were utilized for FTIR runs using a 27 Bruker Tensor analyzer. The precision levels were modified by 4 cm^{-1}.

Thermal gravimetric analysis (TGA)

TGA of TPA-CH POP along with TPA-CH POP-SH nanocomposite carried out over N_2 fluid utilizing the TA Q-50 apparatus. A locked Pt cane utilized as the specimen container, afterwards the ambient temperature was pushed up to 800 °C with a gradient of 20 °C min^{-1} and a median N_2 stream of 50 ml min^{-1}.

Solid state nuclear magnetic resonance (SSNMR)

The Bruker Avance 400 NMR detection system, attached to the Bruker magic-angle whirling (MAS) instrument, served for collecting SSNMR patterns over a period of 32,000 runs.

Surface area as well as porosity aspects

Micromeritics ASAP 2020 surface area and porosity analyzers were utilized to evaluate the surface area along with porosimetry of TPA-CH POP and TPA-CH POP-SH nanocomposite. The use of an ultrapure N_2 flow (up to around 1

atm) and a liquefied N_2 immersion enabled the acquisition of nitrogenic isotherms much easier.

X-ray photoelectron spectroscopy (XPS) bands

Thermo Fisher Scientific ESCALAB 250 utilised a tiny monochromatic Al Kα X-ray laser (15 kV) and a dual-focusing entire 180°spheric sectoral electron scanner to conduct XPS investigations.

Field emission scanner electron microscope (FE-SEM)

The JEOL JSM-7610F SEM is utilized to visualize FE-SEM information. For instance, TPA-CH POP or TPA-CH POP-SH nanocomposite got sputtered with Pt lasting 150 s to ensure explicit vision.

Transmission electron microscopy (TEM)

Upon demonstrating the TPA-CH POP or TPA-CH POP-SH nanocomposite to 200 KV, TEM representations were performed using a JEOL-2100 electron microscope with a scanner.

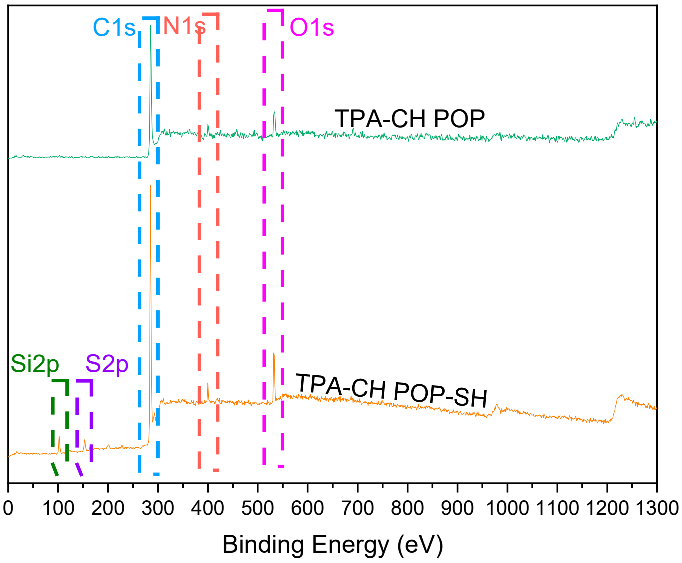

Figure S1 Wide XPS scan of the TPA-CH POP, and TPA-CH POP-SH nanocomposite.

Table S1. Fitting data of TPA-CH POP, and TPA-CH POP-SH nanocomposite

**Element**	**Status**	**Binding Energy (eV)**	**TPA-CH POP**		**TPA-CH POP-SH**	
			FWHM	Area	FWHM	Area
**C1s**	C-N	286.3	1.49	55.17	1.68	883.385
	C-OH	285.5	1.57	133.69	2.69	1646.737
	C=C	284.9	1.17	135.08	1.55	4652.043
	C-Si	283.7			0.87	2391.457
	C-C	283.1			0.83	1788.341
**N1s**	N-C	400.4	1.66	18.437	1.33	608.360
**O1S**	O-H	534.4	1.34	27.8516	1.05	136.381
	HO-C	533.4	1.46	42.187	1.9	418.742
	C=O, Si-O	532.1	1.06	12.674	1.16	948.608
	Crystal Lattice	530.7			1	529.670

	O defect			
**S2p**	S-O 2p1/2	168.5	5.32	11.52
	S-O 2p1/2	166.5	1.59	11.838
	S-C 2p3/2	163.8	2.1	71.553
	S-C 2p1/2	162.6	1.498	92.567
**Si2p**	Si-O	101.15	1.61	629.995
	Si-C	100.05	1.78	113.091

Table S2. CO_2 and N_2 uptakes, CO_2/N_2 selectivity, and isosteric heat of the designed TPA-CH POP, and TPA-CH POP-SH nanocomposite

Sample	**CO_2 uptake ($cm^3\ g^{-1}$)**		**N_2 uptake ($cm^3\ g^{-1}$)**		**Selectivity CO_2/N_2**		**Q_{st} of CO_2 uptake ($KJ\ mol^{-1}$)**	
	298 K	273 K	298 K	273 K	298 K	273 K	0.4 bar	0.15 bar
TPA-CH POP	12.50	18.66	1.67	1.88	1.677	1.882	12.5	18.66
TPA-CH POP-SH	32.69	48.07	2.91	4.05	2.916	4.058	32.69	48.07

Table S3. Comparative study of TPA-CH POP and TPA-CH POP-SH for CO_2 adsorption with earlier reported porous materials.

Sample	CO_2 uptake (mmol/g)		Ref.
	273 K	298 K	
RLF-500	3.13	--	[1]
ELF6	3.29	--	[2]
ELF46	2.46	--	[2]

PECONF-1	1.86	1.34	[3]
PECONF-2	2.85	1.98	[3]
PECONF-4	2.95	1.96	[3]
BPOP-1	1.79	0.98	[4]
BPOP-2	1.45	0.67	[4]
COF-102	1.56	--	[5]
Fc-CMP-1	1.45	--	[6]
BoxPOP-1	--	0.91	[7]
BoxPOP-2	--	1.04	[7]
BoxPOP-3	--	0.29	[7]
$Co_3(BTB)_2(DMA)_4$	--	0.678	[8]
TPA-CH POP	0.83	0.55	This study
TPA-CH POP-SH nanocomposite	2.14	1.45	

1. Hao, G.P.; Li W.C.; Qian D.; Lu A.H. Rapid synthesis of nitrogen-doped porous carbon monolith for CO2 capture. *Adv. Mater.,* **2010**, 22, 853-857.
2. Shi, W.; Zhang X.; Ji Y.; Zhao Z.; Li W.; Jia X. Sustainable preparation of bio-based polybenzoxazine resins from amino acid and their application in CO2 adsorption. *ACS Sustain. Chem. Eng. ACS,* **2019**, 7, 17313-17324.
3. Mohanty, P.; Kull L.D.; Landskron K. Porous covalent electron-rich organonitridic frameworks as highly selective sorbents for methane and carbon dioxide. *Nat. Commun,* **2011**, 2, 401.
4. Sun, X.; Li J.; Wang W.; Ma Q. Constructing benzoxazine-containing porous organic polymers for carbon dioxide and hydrogen sorption. *Eur. Polym. J.,* **2018**, 107, 89-95.

5. Furukawa, H.; Yaghi O.M. Storage of hydrogen, methane, and carbon dioxide in highly porous covalent organic frameworks for clean energy applications. *J. Am. Chem. Soc.,* **2009**, 131, 8875-8883.

6. Li, G.; Liu Q.; Liao B.; Chen L.; Zhou H.; Zhou Z.; Xia B.; Huang J.; Liu B. Synthesis of novel ferrocene-based conjugated microporous polymers with intrinsic magnetism. *Eur. Polym. J.,* **2017**, 93, 556-560.

7. Xu, S.; He J.; Jin S.; Tan B. Heteroatom-rich porous organic polymers constructed by benzoxazine linkage with high carbon dioxide adsorption affinity. *J. Colloid Interface Sci.,* **2018**, 509, 457-462.

8. Kim, D.; Song X.; Yoon J.H.; Lah M.S. 3, 6-Connected metal–organic frameworks based on triscarboxylate as a 3-connected organic node and a linear trinuclear Co3 (COO) 6 secondary building unit as a 6-connected node. *Cryst. Growth Des.,* **2012**, 12, 4186-4193.
